# Comparative Diagnostic Techniques for Cryptosporidium Infection

**DOI:** 10.3390/molecules19022674

**Published:** 2014-02-24

**Authors:** Beauty E. Omoruyi, Uchechukwu U. Nwodo, Chukwuneke S. Udem, Francis O. Okonkwo

**Affiliations:** 1 Department of Biochemistry and Microbiology, University of Forth Hare, Private Bag X1314, Alice 5700, South Africa; E-Mail: sayhi2sa@yahoo.com; 2 Department of Veterinary Physiology and Pharmacology, University of Nigeria, Nsukka 410002, Nigeria; E-Mail: samueludem@gmail.com; 3 Department of Biochemistry, University of Nigeria, Nsukka 410002, Nigeria; E-Mail: obioraokonkwo@gmail.com

**Keywords:** cryptosporidiosis, Cryptosporidium species, staining techniques, antigen detection ELISA, PCR

## Abstract

Diarrhoea caused by Cryptosporidium is usually mild in immune competent individuals but severe in the young and those with underlying disease leading to compromised immunity. The conventional diagnosis of Cryptosporidium requires observation of the infective oocysts however, their tiny size yields indistinct results, thus limiting the effectiveness of the conventional diagnostic technique, modified Ziehl-Neelsen (ZN) differential staining. Consequent to the abovementioned limitation, ZN staining, sandwich antigen detection enzyme linked immunosorbent assay (sad-ELISA) and a direct polymerase chain reaction (PCR) assay techniques were evaluated for diagnostic efficacy. Stool samples were collected from 180 consenting adult patients attending outpatient and inpatient clinics at Victoria Hospital, Alice, Eastern Cape Province of South Africa. Subjects were stratified as; 35 HIV-positive and diarrhoeagenic, 125 HIV-negative diarrhoeagenic and 20 apparently healthy controls. Cryptosporidium incidence following diagnostic techniques were 13 (37.1%; ZN staining), 26 (74.3%; sad-ELISA) and 23 (65.7%; PCR), respectively, among HIV-positive diarrhoeagenic patients and 34 (27.2%; ZN staining), 96 (76.8%; sad-ELISA) and 89 (71.2%; PCR) among HIV-negative diarrhoeagenic patients. Sensitivity, specificity and predictive values of the diagnostic techniques’ efficiency were: sensitivity: 46.2% (HIV-positive) and 32.3% (HIV-negative) against the ZN technique and 96.9% against sad-ELISA and PCR, respectively, for both HIV-positive and -negative patients; specificity was 88.9% (HIV-positive) and 96.6% (HIV-negative) against the ZN technique. Lastly, the predictive values were 92.3% (HIV-positive) and 96.9% (HIV-negative), respectively, following ZN staining. The sad-ELISA technique proved more suitable for the determination of the presence of Cryptosporidium oocysts. The high incidence of Cryptosporidium in HIV-positive subjects as compared to the HIV-negative population accentuates the significance of cryptosporidiosis diagnosis in the treatment and management of HIV cases.

## 1. Introduction

Cryptosporidiosis in immune-compromised, children and the elderly presents severe symptoms. Approximately 30% of the adult population in developed economies and about 60% in developing countries have serologic evidence of prior infection with this organism [1], however, only a handful of people are diagnosed with clinical disease, consequently Cryptosporidium testing is imperative in diarrhoeagenic patients as this will provide a clearer picture of the prevalence of the infection in the various geographical regions of the World.

A majority of cryptosporidiosis patients are symptomatic, with various degrees of diarrhoea characterized by the bulk presence of Cryptosporidium oocysts in their stools. An exhibition of transient self-limiting gastrointestinal disturbance leading to diarrhoea occurs in these symptomatic cases. However, in immunocompromised and malnourished individuals, children and the elderly, infections may be severe and in some cases chronic and life threatening [2]. Diagnosis of the infection generally requires the observation of the infective stage of oocysts, which are usually 4–6 µm in size. Due to the tiny size of these oocysts, differential staining using the modified Ziehl-Neelsen technique and wet mount preparation methods have limited value for the detection of Cryptosporidium in faecal samples where oocysts can easily be confused with other materials present in the samples [2]. In asymptomatic patients or patients with minimal symptoms, the use of routine methods such as sugar flotation concentration and modified acid-fast stains may be insufficient to demonstrate the presence of the parasites. Cryptosporidium are usually not detected during direct examination of specimens in the absence of special stains however, monoclonal antibodies (for the immunosorbent-antigen test ELISA) are used in standard assays in clinical laboratories. Compared to antigen detection, the sensitivity of stool examination by acid-fast staining remains poor and requires an oocyst concentration of over 500,000 per mL in formed stools [3], with fewer cases being detected by acid-fast staining than by immunosorbent assay. Enzyme-linked immunosorbent assays (ELISAs) have been reported to be up to 10 times more sensitive than acid-fast staining [4], making the ELISA method currently the “goldstandard” for antigen detection in infected stool samples [5].

Methods based on microscopic examination are being replaced with techniques which rely on molecular recognition specific for a target pathogen. PCR techniques have the advantages of improved sensitivity and specificity, however these methods have limited applicability at point-of-care or low-resource settings due to their costs, infrastructure needs, and the high technical expertise involved. PCR was used to detect Cryptosporidium in clinical samples, and a 20% prevalence in the stool samples of children with diarrhoea in South African and three-fourths of Ugandan AIDS patients with diarrhea was observed [6,7,8]. Sensitivity of 97%–100% and specificity of 100% have been reported for diagnosis of Cryptosporidium by PCR [9,10].

This study was conducted because of the increasing documentation of infection by Cryptosporidium and the fact that it regularly eludes diagnosis in hospital laboratories. This underdiagnosed nature of Cryptosporidium in diarrhoea cases, coupled with the high prevalence of HIV, which may lead to compromised immunity, has been the motivating factor behind this study undertaken in Nkonkobe Municipality (East Cape Province of South Africa). Given the low sensitivity of microscopy and the varying sensitivity and specificity of ELISA antigen detection, our study aimed to evaluate and compare the use of a staining technique (ZN), ELISA antigen detection and a PCR assay for the detection of Cryptosporidium in HIV-positive and HIV-negative diarrhoea patients. Among patients who have HIV and in whom cryptosporidiosis can be detected early, improvement in immune function with effective antiretroviral therapy can result in dramatic improvement in diarrhea too. The early detection of Cryptosporidium in HIV-positive patients may further add to the knowledge for clinical management of the disease.

## 2. Results and Discussion

### 2.1. Demographic Information of Study Subjects

Demographic characteristics of subjects were 30.6% males and 69.4% females, stratified as shown in Table 1. The population age ranged between 18 to 95 years, with the 57 to 69 age group (17.2%) accounting for majority of female subjects and the 31 to 43 years group (7.2%) being the majority among male subjects, respectively.

**Table 1:** Demographic characteristics of study subjects with respect to sex and age distribution.

	Sex	HIV-Positive Subjects (%)	HIV-Negative Subjects (%)	Control Subjects (%)
		Males 11 (6.1)	Males 34 (18.9)	Males 10 (5.6)
Age (years)		Female 24 (13.3)	Females 91 (50.6)	Female 10 (5.6)
18–30		2 (1.1)	8 (4.4)	2 (1.1)
31–43		13 (7.2)	34 (18.9)	8 (4.4)
44–56		5 (2.8)	11 (6.1)	4 (2.2)
57–69		10 (5.6)	31 (17.2)	1 (0.56)
70–82		3 (1.7)	25 (13.9)	3 (1.7)
83–95		2 (1.1)	16 (8.9)	2 (1.1)

### 2.2. Detection of Cryptosporidium Oocysts: Microscopic Technique

**Figure 1:**
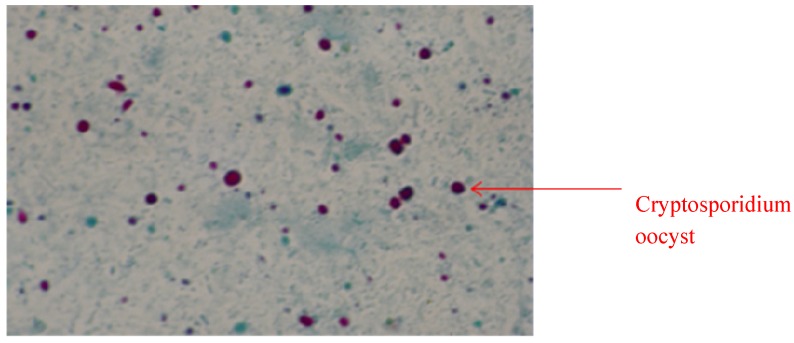
Cryptosporidium oocysts stained pinkish red (examined at 200–400×; Zeiss Axioscop epi-fluorescent microscope) and were observed as thick walled spherical structure of approximately 2–6 µm in diameter.

Microscopic examination of the stool samples of the test population (35 HIV-positive diarrhoeagenics, 125 HIV-negative diarrhoeagenics and 20 apparently healthy control subjects) after modified Ziehl-Neelsen staining, showed the presences of Cryptosporidium oocysts in 26.1% of the population (47 individuals). The positively identified population was stratified as 27.7% HIV-positive diarrhoeagenics and 72.3% HIV-negative diarrhoeagenics. Oocysts of Cryptosporidium seemed to show a spherical morphology with an indistinct internal structure (Figure 1).

Recognition of oocyst morphological features via microscopy, after ZN staining, is the convention in the diagnosis of cryptosporidiosis, however, this techniques is laborious and less sensitive and thus prone to error [10]. Cryptosporidium oocysts are quite tiny, and consequently can easily be mistaken in stool debris as an artifact. Also, they may be easily be confused with other oocysts, such as those of *Cyclospora* species and yeast cells [2]. Conversely, this method would not differentiate between Cryptosporidium species oocysts because they similarly takes up a red to pink colour as do other faecal components, which is the shortfall of this technique compared to others. However, it is cheap and affordable, hence resource-poor countries still rely on the technique as has been variously reported. From Peru, Vitaliano *et al*. [11] reported 29.6% incidence of Cryptosporidium, while a 3.4% incidence was similarly reported by Nagamani *et al*. [12] in India. Other accounts includes 2.7% from Tunisia [13], 12% from Chennai [14], 2.2% from Malaysia, 50% from Zambia, 2.2% and 37.3% from South Italy and France, respectively [15,16,17,18,19]. Resource-rich economies are not left out as this technique was used to report incidence in HIV-positive diarrhoeagenic individuals. Additionally, the ZN technique showed a 27.2% incidence in the study population which cannot be said to corroborate other studies as a prevailing factor in a population affects incidence at that particular point in time. Sethi *et al*. [20] reported an incidence of 0.06% in adults from Chandigarh and 1.5% from Pondicherry [21], all in India.

### 2.3. Detection of Cryptosporidium Oocysts: ELISA Assay Technique

The Sandwich Enzyme Linked Immunosorbent Assay (sad-ELISA) technique employed in the detection of Cryptosporidium infection was positive for 14.4% out of 19.4% representing the HIV- positive subjects (35) and 53.3% out of 69.4% of the HIV-negative subjects, respectively. However, further stratification of the population with respect to sex and HIV status showed males and females to constitute 5% and 9.4%, respectively, out of the 14.4% HIV-positive subjects while the HIV-negative subjects grouped as males and females accounted for 30.6% and 22.8%, respectively.

The ELISA technique showed an incidence as high as 76.8% within the HIV-negative and 74.3% in the HIV-positive population, thus yielding a higher incidence when compared to other techniques. This observation is in accord with earlier reports from the Mexican/US border indicating ELISA positivity of 86.0% cryptosporidiosis in both immunocompromised and immunocompetent individuals [22]. Cryptosporidium antigen detection by ELISA showed a sensitivity as high as 92.3% in HIV-positive subjects, however this technique does not tell us if an infection is active, passive or the antigen presence is as a result of a previous infection [23]. Remarkably, control subjects were all negative to Cryptosporidium antigen. The pattern of result shown by sad-ELISA corroborates reports supporting the claim that fluorescent monoclonal methods increase the sensitivity and specificity of Cryptosporidium oocyst detection and hence provide excellent screening techniques and offer useful data for epidemiological studies, and consequently, control of the parasite [24,25].

### 2.4. Detection of Cryptosporidium Oocysts: PCR Amplification of Genomic DNA

**Figure 2:**
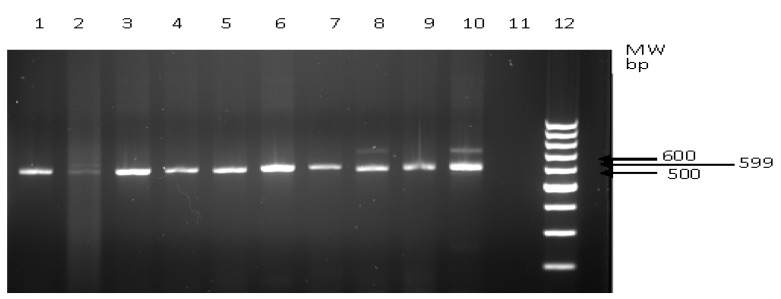
PCR Amplification products SSU rRNA gene from cDNA. Lane 12 = 100 bp ladder; lane 11= negative control.

One hundred and twelve (112) stool samples representing 62.2% of the study population showed a small subunit (SSU) rRNA gene of the expected size (599 bp) indicating the presence of Cryptosporidium (Figure 2). SSU rRNA gene amplicons were not detected in the negative control as shown in lane 11 of the gel. Similarly, the control subjects did not show the presence of SSU rRNA gene. The PCR technique showed a high incidence however, it trailed behind the ELISA technique in this respect. The detection of Cryptosporidium SSU rRNA gene by PCR cannot account however for the active or passive status of infection‒it simply indicates presence of Cryptosporidium in the test subject. Notwithstanding the variation in incidences recorded with the ELISA and PCR techniques, statistically there was no significant difference in sensitivity and specificity using both techniques. Bialek *et al.* [10] and Weitzel *et al*. [26] reported incidences using both techniques and similarly noted that differences between them were not significant.

### 2.5. Comparative Evaluation of Cryptosporidium Detection Techniques

Comparative evaluation of the diagnostic techniques showed percentages of detection as follows: 74.3% (sad-ELISA), 65.7% (PCR) and 37.1% (ZN) against HIV-positive subjects and 76.8% (sad-ELISA), 71.2% (PCR) and 27.2% (ZN) against HIV-negative subjects, respectively (Table 2).

**Table 2:** Sensitivity, specificity, positive predictive value and negative predictive value of the techniques.

TDiagnostic Techniques	HIV status	Cryptosporidium Incidence (%)	Sensitivity (%)	Specificity (%)	Positive Predictive Value (%)	Negative Predictive Value (%)
ZN	Positive	13/35 (37.1)	46.2	88.9	92.3	36.4
	Negative	34/125 (27.2)	32.3	96.6	96.9	30.1
sad-ELISA	Positive	26/35 (74.3)	92.3	36.4	46.2	88.9
	Negative	96/125 (76.8)	96.9	30.1	32.3	96.9
PCR	Positive	23/35 (65.7)	75.0	42.1	52.2	66.7
	Negative	89/125 (71.2)	96.9	32.6	79.5	77.8

Furthermore, the sensitivity of the techniques ranged from 32.3% to 96.9% with sad-ELISA and PCR techniques showing the highest sensitivity at 96.9% each with HIV-negative test subjects. Conversely, the ZN technique showed higher specificity with 96.6% and 88.9%, respectively, against HIV-negative and -positive subjects, respectively. The other two techniques, sad-ELISA and PCR, had low values ranging 30.1% to 42.1% (Table 2). The control subjects (20 apparently healthy subjects) were negative for Cryptosporidium according to all three techniques.

The positive predictive values for HIV-positive subjects ranged from 46.2% (sad-ELISA) to 92.3% (ZN), while HIV-negatives were 32.2% (sad-ELISA) and 96.9% (ZN), respectively. Additionally, negative predictive values for HIV-positive subjects ranged 36.4% (ZN) to 88.9% (sad-ELISA), while HIV-negative subjects ranged from 30.1% (ZN) to 96.9% (sad-ELISA), as shown in Table 2.

Variations in sensitivities and specificities are understandable because the sad-ELISA detects antigens of the pathogen which may have been from previous infections and/or an active infection. Likewise the PCR technique picks up genomes of both infective and non-infective Cryptosporidiumoocysts, thus greatly increasing the sensitivity of both diagnostic techniques. Conversely, specificity is higher with the ZN technique as mature active oocysts are mostly detected. Furthermore, the variation in the results with different test subject groupings may have been influenced by their previous encounters with the pathogen.

## 3. Experimental

### 3.1. Patients and Control Subjects

Stool samples were collected once-off from 180 adults attending outpatient and inpatients clinics in Victoria Hospital over a period of eighteen months. The study population was stratified as 35 HIV-positive diarrhoeagenics, 125 HIV-negative diarrhoeagenics and 20 apparently healthy individuals without any history suggestive of cryptosporidiosis who were sent to the hospital for investigation. Clinical information of each patient as well as any drugs and chemotherapeutics used for treatment by each patient was obtained, with their full consent to participate in the study. Additional patient information was obtained by means of a questionnaire. Confidentiality of the voluntary participants was maintained.

### 3.2. Stool Specimens and Processing

Stool samples were collected in the laboratory facilities at Victoria Hospital and transported in cool wide mouthed plastic containers to the laboratory in the Department of Microbiology and Biochemistry at the University of Fort Hare, Alice, South Africa. A portion of each sample was kept at −20 °C for antigen detection and PCR analysis while the remainder was used for microscopic analysis.

### 3.3. Microscopic Examination of Cryptosporidium Oocysts

Stool samples were scooped and smeared on a clean glass slide, followed by fixing with 95% absolute methanol and stained using the modified Ziehl-Neelsen stain and air dried. Afterwards the smear was further stained with cold carbol fuchsin and allowed to stand for 10 minutes after which it was washed off with clean tap water. The smear was decolorized with 3% hydrochloric acid (HCl) in 95% ethanol, rinsed off and counterstained with 0.25% weight per volume malachite green for 30 s. The smear was, again, washed off with clean tap water and air dry. The slide was then observed microscopically for oocysts [24].

### 3.4. Cryptosporidium Specific Antigen Detection

Cryptosporidium-specific antigens were detected in stool samples using a fluorescent monoclonal antibody test (MOCI Ltd, West Sussex, UK) in accordance with manufacturer’s instructions. ELISA microtiter plates were coated with fluorescent monoclonal antibody; 300 µL SupperBlock^®^ blocking buffer (Antimicrobial Agent, Rockford, IL, USA) was used to wash each well thrice and emptied afterwards by plate inversion. Plates were dried, sealed in plastic bag and stored at 4 °C until used.

Supernatant of fecal suspension was added to the wells of the microtiter plate pre-coated with fluorescent monoclonal antibodies specific for Cryptosporidium antigen. Two hundred µL of horseradish peroxidase (HRP)-labelled mouse immunopure 1gG monoclonal anti-CSA conjugate was added to each well of the plate, covered, and incubated for 60 minutes at 20 °C with shaking. The plates were washed five times with buffer to remove unbound antibody conjugate. A colourless single-component enzyme substrate (tetramethylbenzidine-TMB) was added, the plates incubated for 10 min at 20 °C and observed for a colour change. A stop solution was added and the optical density (OD) was read on an ELISA plate reader (Model 680, Bio-Rad Laboratories, Hercules, CA, USA) at an absorbance of 450 nm. Cryptosporidial antigen was used as a positive control. The cut-off value for a positive reaction was calculated to be double the optical density value of the negative control. OD values >0.05 were considered positive following the manufacturer’s guidelines.

### 3.5. PCR Assay for Cryptosporidium

DNA extraction: Cryptosporidium DNA was extracted following the Zymo-Research (ZR) protocol; about 200 mg of faecal sample was added to 750 µL lysis buffer in a ZR bashing bead lysis tube, the mixture was homogenized and centrifuged (12,000 rpm; 1 min), after which 400 µL of the supernatant was aspirated into another tube and with subsequent centrifugation (7,000 rpm; 1 min). The filtrate was mixed with 1,200 µL of faecal DNA binding buffer afterwards, 800 µL of the mixture was transferred to a new Zymo-spin 11C column in a collection tube and centrifuged (10,000 rpm; 1 min). The step was repeated after which 200 µL DNA pre-wash buffer was added and centrifuged (10,000 rpm; 1 min). Next, 500 µL faecal DNA wash buffer was added to the “11C” tubes, centrifuged (10,000 rpm; 1 min) and transferred to a new clean tube of 1.5 mL capacity with subsequent centrifugation (8,000 rpm; 1 min).

### 3.6. PCR Amplification of Cryptosporidium DNA

*Cryptosporidium parvum* 18S rRNA gene of 599 bp was amplified with the following forward abd reverse primers; CPF-5'-GTGCCAGCAGCCGCGGTAAT-3' and CPR-(5'-AAGCCGCAGGCTCCA CTCCT-3') which correspond to the 542 to 561 and 1,140 to 1,121 positions, respectively, on the coding and negative strand of GenBank sequence Af093489. The reactions were performed with a model 9600 Perkin-Elmer thermocycler (Perkin-Elmer, Foster City, Calafonia, CA, USA) in 0.5 mL thin-wall Eppendorf tubes [10]. Each 50 µL PCR tube reaction mixture contained PCR buffer (10 mM Tris-HCl, 50 mM KCl [pH 8.3]), 1.5 mM MgCl_2,_ 200 µM dNTPs, 0.5 µM of each specific oligonucleotide primers, 2.5 U of *Taq* DNA polymerase (Boehringer Mannheim GmbH, Mannheim, Germany), and 5 µL of purified DNA extract. After a fifteen-minute hot start at 95 °C, the reactions went through 35 cycles of denaturation at 94 °C for 1 min, annealing at 65 °C for 1 min, and extension at 72 °C for 1 min, followed by a 72 °C incubation for 15 min for strand completion. An aliquot of each PCR product was examined by agarose gel electrophoresis. Each run included a negative control, consisting of a reaction mixture with water instead of DNA template. Amplified DNA was run in 2% electrophoresis agarose gel containing 0.17 L 1× TAE buffer. Gel electrophoresis at 85 volts for 1.5 h was performed using a Subcell tank (BioRad Laboratories, Hercules, CA, USA) and immersion in 1× TAE buffer. The gel was visualized by 10 µg/mL ethidium bromide (Sigma Chemical Co., St. Louis, MO, USA) under ultra-violet light illumination (FotoPrep R, Fotodyne Inc, New Berlin, WI, USA) to view the anticipated molecular size.

### 3.7. Ethical Clearance

This study was approved by the Institutional Ethics Review Board of the University of Fort Hare through the Govan Mbeki Research Development Centre (GMRDC).

### 3.8. Statistical Analysis

Sensitivity, specificity, predictive value (positive and negative) and diagnostic efficacy of various techniques were determined following the methods of Galen and Gambino [27].

## 4. Conclusions

The ZN staining technique was less sensitive for the detection of Cryptosporidium in the study population, however it has the advantage of being the only technique that only indicates active infections, unlike the ELISA and PCR techniques which may not distinguish between active and non-active infections. Furthermore, less expertise and cost is needed for the application of the ZN staining technique although the use of this technique means that several cases of cryptosporidiosis will go undiagnosed as it less sensitive. A combination of the ZN staining technique with either of the sad-ELISA and PCR techniques would be a “gold standard” as specificity and sensitivity would be very high, thus ensuring Cryptosporidium infections do not go undiagnosed. Lastly, the incidence of Cryptosporidium infection was noticeably higher in HIV-positive individuals, hence the need to monitor Cryptosporidium oocysts in HIV-positive individuals to help provide more effective therapy.

## References

[1] Cheng A.C., McDonald J.R.M.D., Nathan M. (2005). Infectious diarrhoea in developed and developing countries. J. Clini. Gastroenterol..

[2] Connelly J.T., Nugen S.R., Borejsza-Wysocki W., Durst R.A., Montagna A.J. (2008). Human pathogenic Cryptosporidium species bioanalytical detection method with single oocyst detection capability. Anal. Bioanal. Chem..

[3] Weber R.R.T., Bryan H.S., Bishop S.P., Wahlquist J.J., Sullivan D.D. (1991). Threshold of detection of Cryptosporidium oocysts in human stool specimens: evidence for low sensitivity of current diagnostic methods. J. Clin. Microbiol..

[4] Katanik M.T., Schneider S.K., Rosenblatt J.E., Hall G.S., Procop G.W. (2001). Evaluation of Color PAC Giardia/Cryptosporidium rapid assay and ProSpecT Giardia/Cryptosporidium microplate assay for detection of Giardia and Cryptosporidium in fecal specimens. J. Clin. Microbiol..

[5] Chappell C.L., Okhuysen P.C. (2002). Cryptosporidiosis. Curr. Opin. Infect. Dis..

[6] Higgins J.A., Fayer R., Trout J.M., Xiao L., Lal A.A., Kerby S., Jenkins M.C. (2001). Real-time PCR for the detection of *Cryptosporidium parvum*. J. Microbiol. Meth..

[7] Samie A., Bessong P.O., Obi C.L., Sevilleja J.E., Stroup S., Houpt E., Guerrant R.L. (2006). Cryptosporidium species: Preliminary descriptions of the prevalence and genotype distribution among school children and hospital patients in the Venda region, Limpopo Province, *South Afr.*. Exp. Parasitol..

[8] Tumwine J.K., Kekitiinwa A., Nabukeera N., Akiyoshi D.E., Rich S.M., Widmer G., Feng X., Tzipori S. (2003). *Cryptosporidium parvum* in children with diarrhoea in Mulago Hospital, Kampala, Ugand. Am. J. Trop. Med. Hyg..

[9] Morgan U.M., Pallant L., Dwyer B.W., Forbes D.A., Rich G., Thompson R.C. (1998). Comparison of PCR and Microscopy for detection of *Cryptosporidium parvum* in human faecal specimens: Clinical trial. J. Clin. Microbiol..

[10] Bialek R., Binder N., Dietz K., Joachim A., Knobloch J., Zelck U.E. (2002). Comparison of fluorescence, antigen and PCR assays to detect *Cryptosporidium parvum* in faecal specimens. Diagn. Microbiol. Infect. Dis..

[11] Galen R.S., Gambino S.R. (1975). Beyond Normality: The predictive value and efficiency of medical diagnosis. J. Infect. Dis..

[12] Vitaliano A.C., Caryn B., Jacqueline R., Lillia C., Charles R.S., Ynes O., Robert H., Lihua X. (2008). Cryptosporidium species and subtypes and clinical manifestation in children in Peru. Emerg. Infect. Dis..

[13] Nagamani K., Pavuluri P.R., Guaneshwari M., Prasanthi K., Raomi N.K. (2007). Molecular characterization of Cryptosporidium. An emerging parasite. Indian J. Med. Microbiol..

[14] Rym E., Mohamed M., Karim A., Rim A., Fethi M., Fakher K., Fracis D., Alda B. (2007). Identification of Cryptosporidium species infecting humans in Tunisia. Am. J. Trop. Med. Hyg..

[15] Kumar S.S., Ananthan S., Sarvanan P. (2002). Role of coccidian parasites in causation of diarrhoea in HIV infected patients in Chennai. Indian J. Med. Res..

[16] Stark D., Fotedar R., Hal S.V., Beebe N., Marriot D., Ells J.T., Harkness J. (2007). Prevalence of enteric protozoa in human immunodeficiency virus (HIV)-positive and HIV-negative men who have sex with men from Sydney, Australia. Am. J. Trop. Med. Hyg..

[17] Oguntibeju O.O. (2006). Prevalence of intestinal parasites in HIV-positive/AIDS patients. Malays. J. Med. Sci..

[18] Brandonisio O., Maggi P., Panaro M.A., Lisi S., Andriola A., Acquafredda A., Angarano G. (1999). Intestinal protozoa in HIV-infected patients in Apulia, South Italy. Epidemiol. Infect..

[19] Chintu C., Luo C., Baboo S., Med M., Khumalo-Ngwenya B., Mathewson J., DuPont H.L., Zumla A. (1995). Intestinal parasites in HIV-seropositive Zambian children with diarrhoea. J. Trop. Paed..

[20] Cotte L., Rabondonirina M., Piens M.A., Perrcard M.M., Trepo C. (1993). Prevalence of intestinal protozoans in French patients infected with HIV. J. Acquir. Immune. Defic. Syndr..

[21] Sethi S., Sehgal R., Malla N., Mahajan R.C. (1999). Cryptosporidiosis in a tertiary care hospital. Natl. Med. J. India.

[22] Parija S.C., Shivaprakash M.R., Jayakeerthi S.R. (2003). Evaluation of lacto-phenol cotton blue (LPCB) for detection of Cryptosporidium, Cyclospora and Isospora in the wet mount preparation of stool. Acta Trop..

[23] Redlinger T.V., Corella-barud J., Graham A., Galindo R., Cardenas V. (2002). Hyperendemic Cryptosporidium and Giadia in households lacking municipal sewer and water on the United State-Mexico border. Am. J. Trop. Med. Hyg..

[24] Casemore D.P. (2004). The antibody response to Cryptosporidium: development of a serological test and its used in a study of immunologically normal persons. J. Clin. Infect..

[25] Xiao L., Herd R.P. (1993). Quantitation of Giardia cysts and Cryptosporidium oocysts in fecal sample by direct immunofluorescence assay. J. Clin. Microbiol..

[26] Alles A.J. (1995). Prospective comparison of direct immunofluorescence and conventional staining methods for detection of Giardia and *Cryptosporidium* spp. in human fecal specimens. J. Clin. Microbiol..

[27] Weitzel T., Dittrich S., Möhl I., Adusu E., Jelinek T. (2007). Evaluation of seven commercial antigen detection tests for Giardia and Cryptosporidium in stool samples. Clin. Microbiol. Infect..

